# Supplementary material for: Evolution of Bacterial Gene Transfer Agents
Source: Front Microbiol. 2018 Oct 25;9:2527. doi: 10.3389/fmicb.2018.02527 (PMC6209664; doi:10.3389/fmicb.2018.02527)

Supplementary Table 1: Parameter Variables used in the text. Definitions of all variables used throughout the text.

**Parameter**	**Description**
μ	Mutation rate per genome per generation
i	Number of mutations in a cell's genome
w	Fitness function
s	Selection coefficient of the fitness function
**α**	Epistasis factor of the fitness function
pGTA	Probability that a GTA+ cell activates GTA genes and produces particles
max	Maximum number of mutations considered
eff	Efficiency of GTA production and recombination

SUPPLEMENTARY FIGURES:

Supplementary Figure 1: Flow chart of the recombination model. The calculations are described in the Methods section of the text: Left panel: Overview of a set of runs. Right panel: details of the steps within each generation of a run.

1. **Calculate DNA_Matrix:** This matrix gives the probabilities (*P*) that DNA fragments of size *r* from cells with *i* mutations will contain *j* mutations. $P = i!/((j! * (i-j)!) * r^{j} * (1-r)^{i-j}$
2. **Selection:** The fraction of each mutation class that survives selection is calculated by a fitness function with the form $w = (1-s)^{i \wedge \alpha}$, where *i* is the number of mutations in the cell's genome and α is an epistasis coefficient that specifies how interactions between mutations affect fitness. Specific fitness functions used are shown in Figure 3.
3. **Production of GTA particles:** The regulation function determines the fraction of each mutation class that may produce GTA particles, the *pGTA* determines the subfractions of these that actually do produce GTAs. The DNA_matrix is applied to these subfractions to get the mutation distribution of mutations in GTA particles. This distribution is randomized about its mean to simulate uptake of multiple short DNA fragments rather than a single long one.
4. **Lysis of GTA producers:** The fraction of cells that produced GTA particles is subtracted from each mutation class.
5. **Recombination:** At each generation the DNA matrix (DNA_matrix) is used to calculate from the GTA distribution a fresh Recombination matrix (Rec_matrix) that gives, for each mutation class, the distribution of changed genotypes after GTA-mediated recombination. Rec_matrix is then applied to the distribution of mutation classes after the lysis step.
6. **Mutation:** The distribution of new mutations in the population is Poisson.

7. **Reproduction:** At the end of each generation the mutation distribution is normalized to compensate for deaths due to selection and lysis.

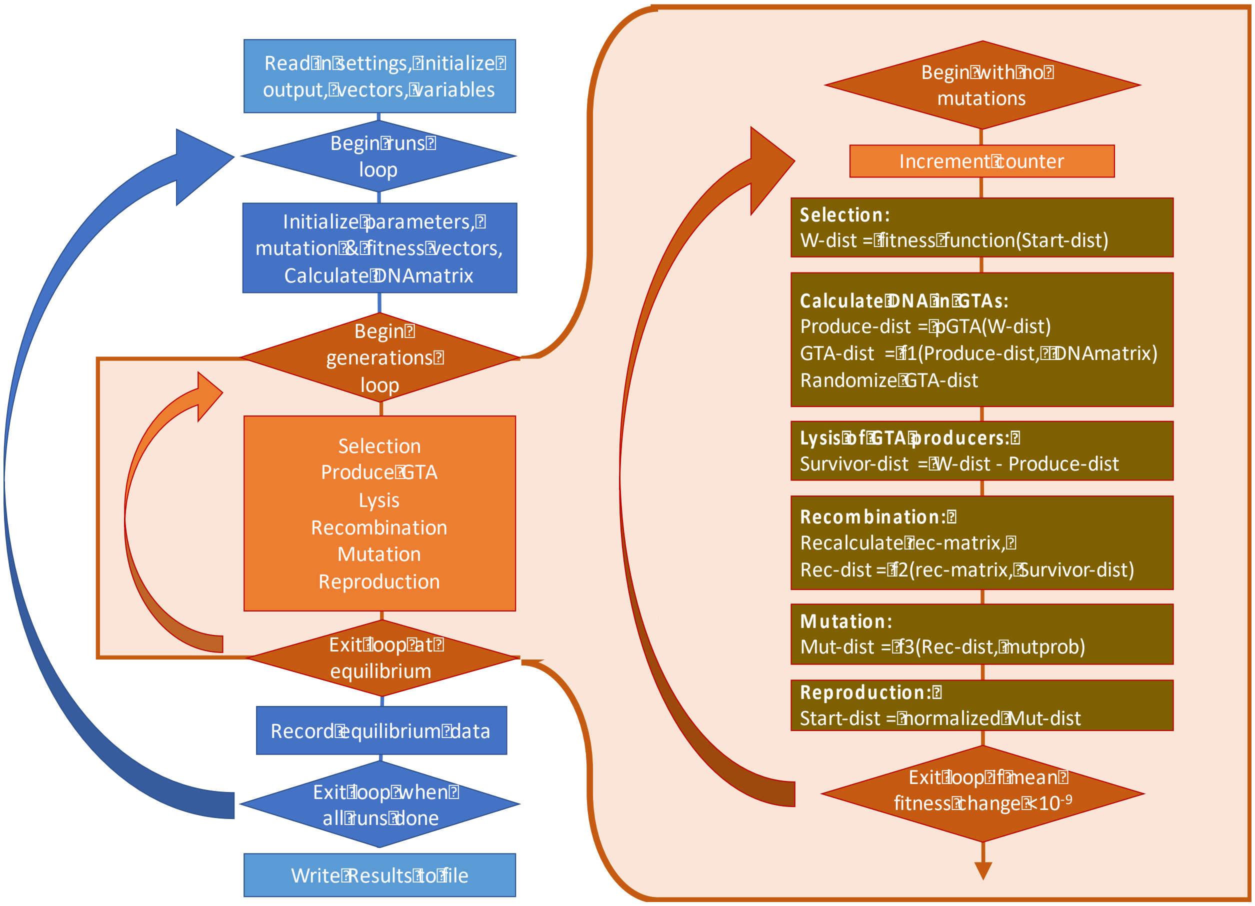

Supplementary Figure 2: Effects of epistasis and recombination on mutation distributions. A. α=0.8. B. α=1.0, C. α=1.2, D. α=1.5. Blue bars: distributions of mutations at equilibrium with no GTA production (no recombination). Green, yellow, grey and orange bars: distributions of mutations at equilibrium when 0.1 of population produces and recombines GTA (no lysis). Vertical arrows and numbers indicate the mean number of mutations per genome in each population.

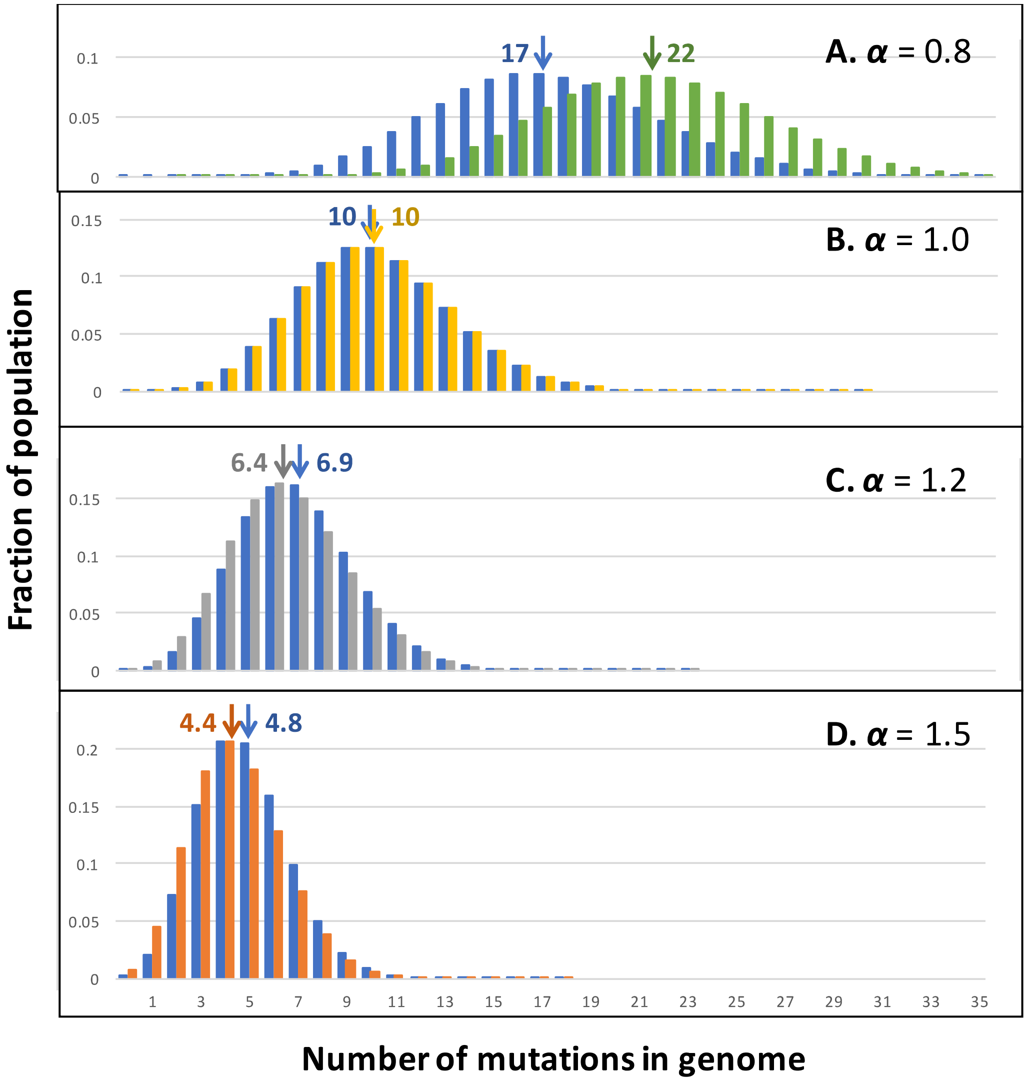

Supplementary Figure 3: Fitness effects of regulating participation in GTA-production and/or GTA-recombination. The first word in each orange box describes regulation of GTA production, the second describes regulation of GTA uptake and recombination. ‘High’: regulation by the High-log function in Fig. 5; ‘All’: no regulation, all cells participate; ‘Low’: regulation by the Low-log function in Fig. 5. Blue lines: simulations without lysis; red lines: simulations with lysis.

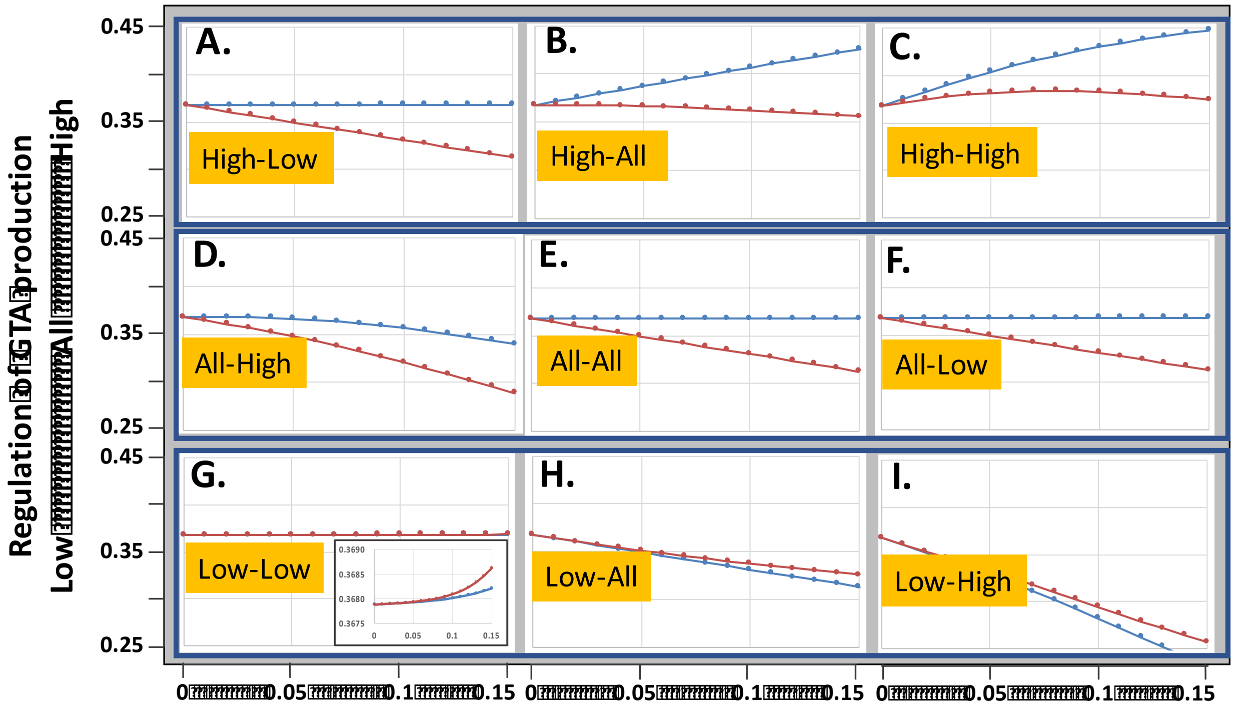

Supplementary Figure 4: Linear and cutoff functions used to limit GTA-production and/or GTA-recombination. Functions are indicated by lines superimposed on a typical equilibrium mutation distribution (pale green bars). Left Y axis: frequency of each mutation class in the population. Right Y axis: Relative probability of producing GTA or recombining. Cutoff functions (black and red): Only all cells with more/fewer mutations than max-class participate. Linear functions (blue and yellow): For cells with more/fewer mutations than max-class, probability of participation is a linear function of mutation difference from mean.

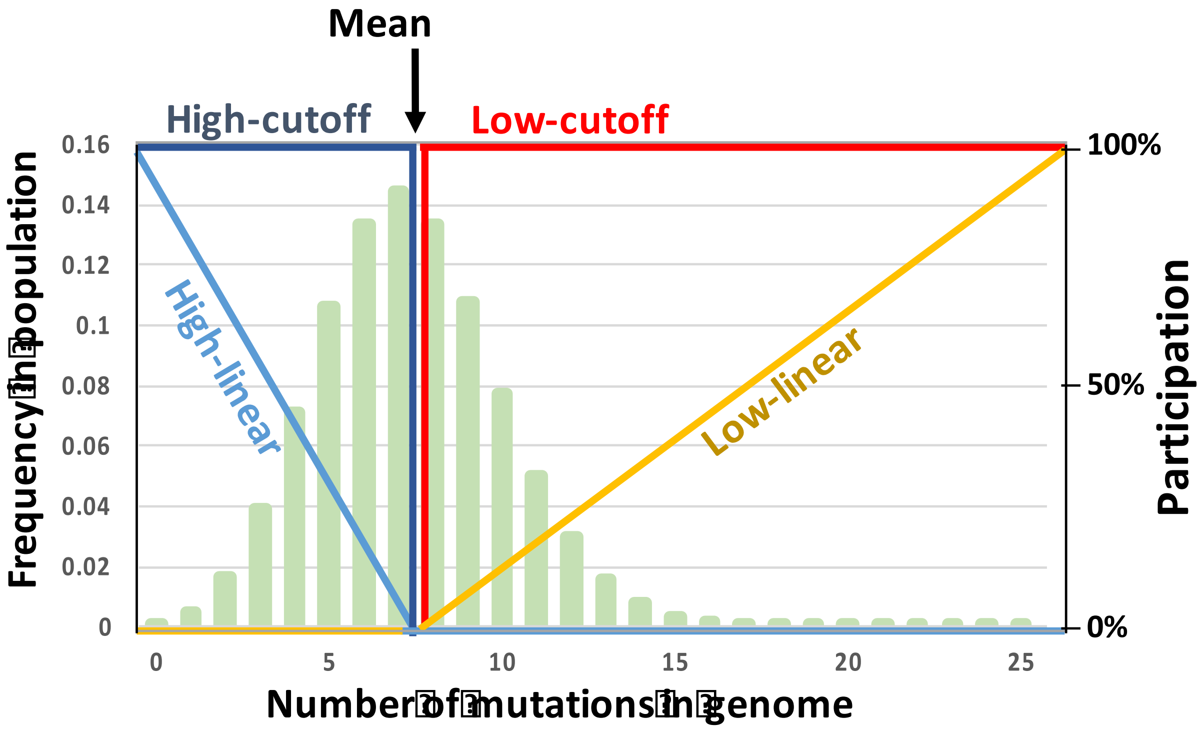

Supplementary Figure 5: Fitness effects of coordinately regulating GTA-production and GTA-recombination. Regulation functions as indicated. Blue: simulations without lysis; orange: simulations with lysis. Panels A-I: all combinations of low-low regulation. Note the very narrow range of the Y-axis. Panels J-R: all combinations of high-high regulation.

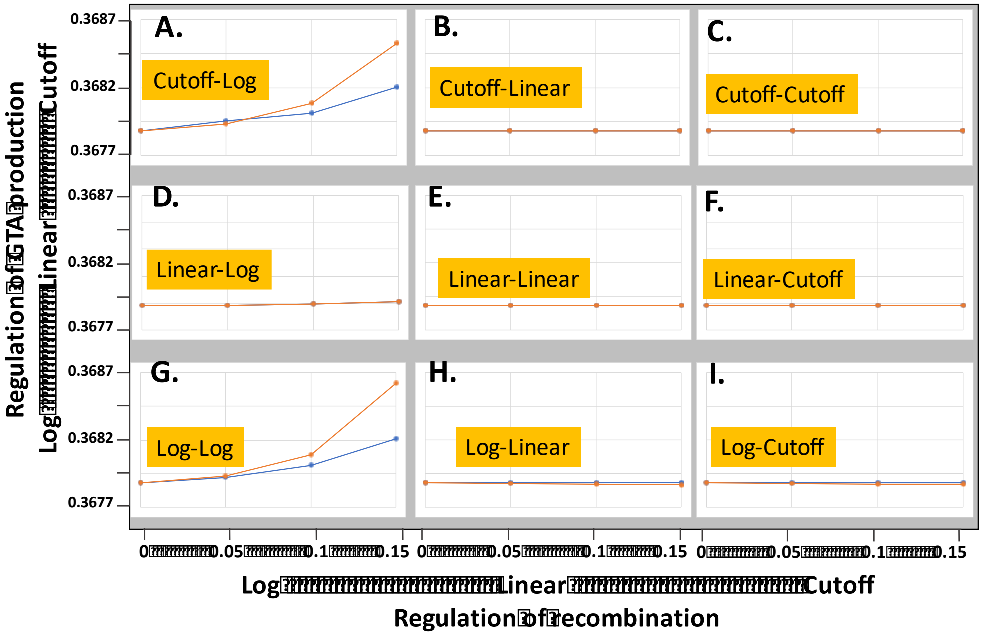

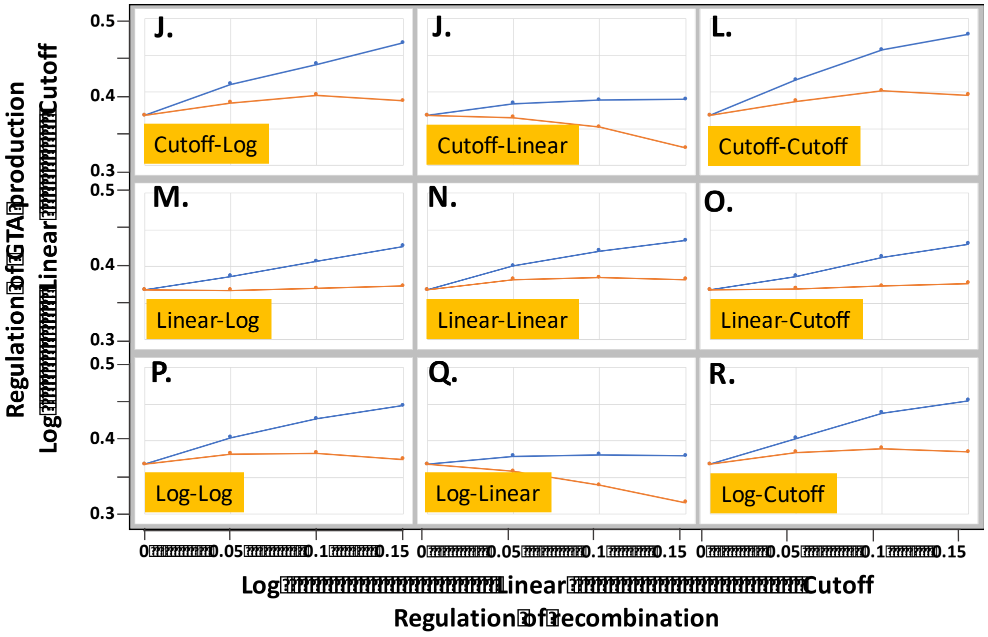

Supplementary Figure 6: Schematic calculation of transduction efficiency. Assume a mixed population of 10,000 cells, 5000 S^R G^S and 5000 S^S G^R. 1% of these cells produce GTA particles with 100% efficiency, so there are 50 S^R particles and 50 G^R particles. Assume 100% efficiency of transduction. Half of these will be into cells of the same genotype as the GTA producer, so this will give 25+25=50 S^R G^R cells, 0.005 of the original population..

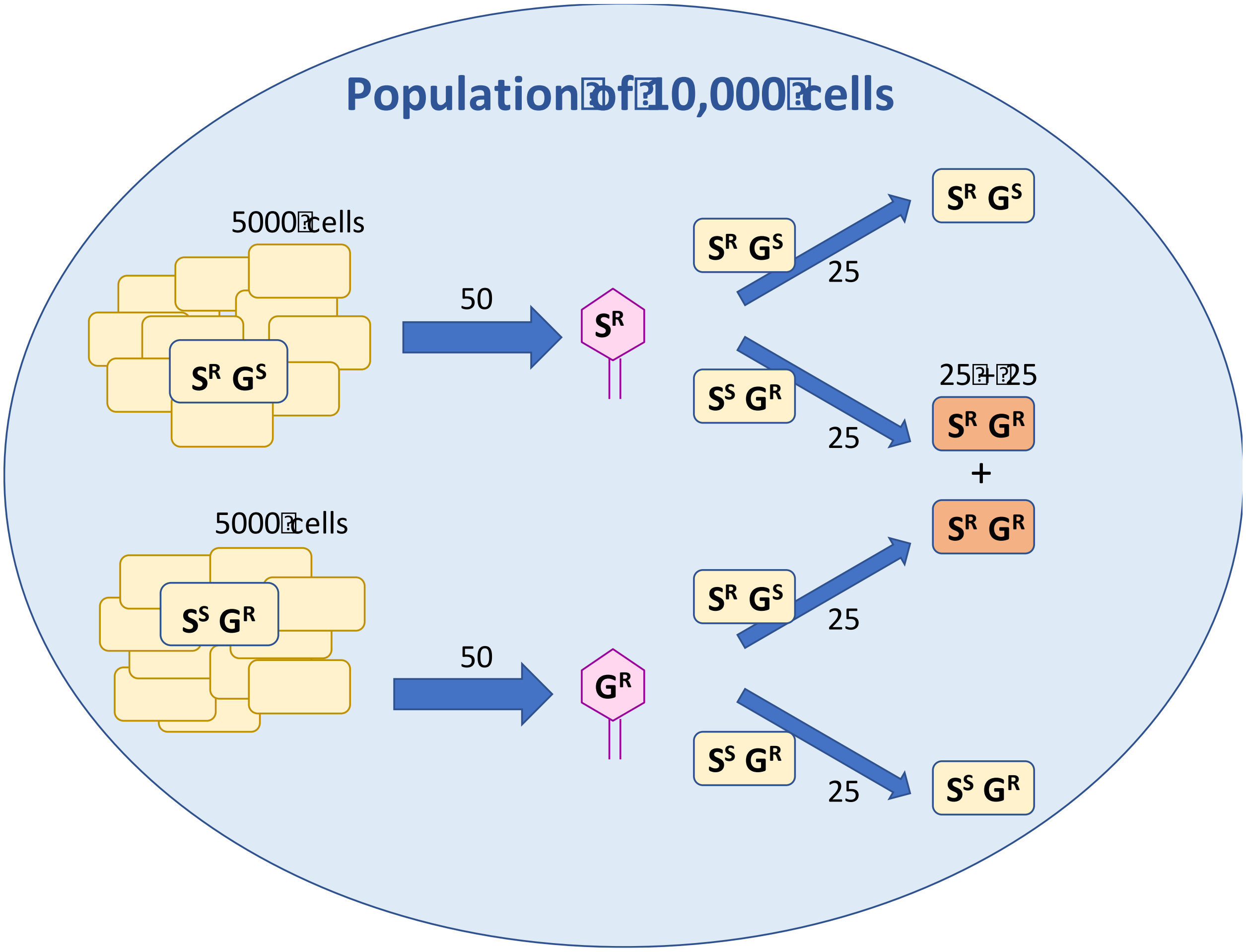

Supplement: Supplementary file 1 [file Data_Sheet_1.pdf]
